# The efficacy and safety of stereotactic body radiotherapy combined with systematic therapy for metastatic renal cell carcinoma: a systematic review and meta‐analysis

**DOI:** 10.1002/mco2.544

**Published:** 2024-04-24

**Authors:** Shiyu Zhang, Xingyu Xiong, Nan Xie, Weitao Zheng, Yongjun Li, Tianhai Lin, Qiang Wei, Ping Tan

**Affiliations:** ^1^ Department of Urology West China Hospital, Sichuan University Chengdu China; ^2^ Emergency Department of West China Hospital, West China School of Nursing, Sichuan university Chengdu China; ^3^ Nursing Key Laboratory of Sichuan Province Sichuan University Chengdu China; ^4^ West China School of Medicine West China Hospital Sichuan University Chengdu China

**Keywords:** metastatic renal cell carcinoma, stereotactic body radiation therapy (SBRT), systemic therapy

## Abstract

There is considerable interest in the potential of stereotactic body radiation therapy (SBRT) combined with systemic therapy such as tyrosine kinase inhibitors (TKIs) or immune checkpoint inhibitors (ICIs). However, its efficacy and safety remain unclear. The purpose of this study was to evaluate the efficacy and safety of conducting SBRT during ICI or TKI treatment in different disease settings for patients with metastatic renal cell carcinoma (mRCC). A total of 16 studies were ultimately included. Under the random effects model, the pooled 1‐year local control rate (1‐yr LCR) and objective response rate (ORR) were 90% (95% confidence interval [CI]: 80%–95%, *I* ^2^ = 67%) and 52% (95% CI: 37%–67%, *I* ^2^ = 90%), respectively. SBRT concomitant with different systemic therapy yield significant different 1‐yr LCR (*p* < 0.01) and ORR (*p* = 0.02). Regarding survival benefits, the pooled 1‐year progression‐free survival (1‐yr PFS) and 1‐year overall survival (1‐yr OS) rates were 45% (95% CI: 29%–62%, *I* ^2^ = 91%) and 85% (95% CI: 76%–91%, *I* ^2^ = 66%), respectively. 1‐yr PFS and 1‐yr OS in different disease settings demonstrated significant difference (*p* < 0.01). As for toxicity, the pooled incidence of grade 3–4 adverse events was 14% (95% CI: 5%–26%, *I* ^2^ = 90%). This study highlights the feasibility of utilizing these strategies in mRCC patients, especially those with a low metastatic tumor burden.

## Graphical abstract

1. SBRT applying during systemic therapy is safe and effective in the management of mRCC.
2. SBRT applied disease setting and timing may be critical for clinical outcomes and needed further research.

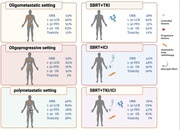

## 1 INTRODUCTION

In 2022, global kidney cancer incidence reached an estimated 431,288 new cases, with a corresponding 179,368 fatalities.^1^ Among all kidney malignancies, renal cell carcinoma (RCC) is the most common type (90%), predominantly including clear cell RCC (ccRCC, 70%), papillary RCC (15%–10%), and chromophobe RCC (5%) histologically.^2^ Overall, 25%−30% of patients present with metastatic disease at time of diagnosis and nearly 50% of RCC patients eventually develop metastatic disease. The brain, lung, liver, and bone are frequent metastasis sites. Metastatic renal cell carcinoma (mRCC) generally resulted in poor outcomes, with more than 5‐year overall survival (OS) ranging from 5% to 10%.^3^

RCC has historically been considered biologically radioresistant and radiotherapy has been largely reserved for palliation purpose. However, in recent years, stereotactic body radiation therapy (SBRT), also termed as stereotactic ablative radiation therapy, has yielded dramatic cytotoxic effects on RCC through the delivery of high dose of radiation in a few fractions. In contrast to conventional radiotherapy, ablative radiation approaches are inclined to trigger cell death via hypoxia mediated by vascular endothelial cells and apoptosis mediated by ceramide. This differs from the conventional mechanism, which typically induces mitotic catastrophe through DNA damage.^4^ The efficacy and safety of SBRT for primary RCC were evaluated by a recent meta‐analysis. The estimated local control rate (LCR) was 97.2%. The estimated incidence of grade 3−4 toxicity was 1.5%, with a 7.7ml/min decrease in estimated glomerular filtration rate (eGFR) after SBRT. The safety and efficacy of SBRT for mRCC have continued to be evaluated in recent years. In oligometastatic setting, metastasis‐directed SBRT achieved similar local control (LC) to surgical metastasectomy,^5^ with a reported 1‐year LC of 90% and any significant toxicity of 1% for both extracranial and intracranial lesions.^6^ In addition to tumor control, providing SBRT for lesions stemming from RCC that have metastasized to the bone and brain can bring about significant alleviation of local symptoms, such as cancer‐related pain.^7^

Except for local therapy of metastases, systemic therapy, such as targeted therapy and immunotherapy or their combination, achieve substantially improved OS for patients with mRCC. According to the European Association of Urology 2023 Guidelines on RCC, first‐line tyrosine kinase inhibitors (TKIs) plus immune checkpoint inhibitors (ICIs) have become the standard of care for all International Metastatic Renal Cell Carcinoma Database Consortium (IMDC) risk patients. Double ICIs combining nivolumab and ipilimumab are recommended for IMDC intermediate/poor risk mRCC patients. Even though systemic therapy demonstrated considerable benefits, few of them have durable clinical response, with a median progression‐free survival (PFS) ranging from 11.2 to 23.9 months in the first‐line treatment. Therefore, many studies have investigated the new combination approach by integrating SBRT with systematic therapy to help overcome drug resistance and realize long‐term LC instead of short‐term palliation.

The combination of SBRT with systemic therapy is increasingly being explored in different mRCC disease settings and aroused considerable interest; however, evidence about the efficacy and safety of combining SBRT with systemic therapy is lacking. Therefore, we conducted a systematic review and meta‐analysis to evaluate the safety and efficacy of combining metastases‐directed SBRT with systematic therapy in patients with metastatic RCC and discussed appropriate scenarios for clinical practice.

## 2 RESULTS

### 2.1 Study characteristics

A total of 15 full studies and one meeting abstracts were ultimately included in this study. Five were retrospectively designed, and nine were prospective clinical trials^8^, ^9^, ^10^, ^11^, ^12^, ^13^, ^14^, ^15^, ^16^, ^17^, ^18^, ^19^, ^20^, ^21^ (Table 1). In total, 596 participants with mRCC were included. The median age ranged from 53 to 66 years. Eastern Cooperative Oncology Group (ECOG) scale or Karnofsky performance status (KPS) were used to evaluate performance status of participants. The majority of patients had ECOG scores ≤2 or KPS scores ≥80, and the most of patients were at intermediate IMDC risk. Most of patients received radical or partial nephrectomy. Most participants had a limited number of metastatic sites (≤5). The majority histology of primary tumor was ccRCC.

**TABLE 1:** Baseline characteristics of included studies.

Author, year	Study design	Sample size	Age (year)	Follow‐up (months)	IMDC risk	Performance status	Nephrectomy	No. of metastases
Miller, 2016	Retrospective	70	‒	15	‒	Median KPS = 80	‒	≤5; 77.1%
De Wolf, 2017	Phase I	13	66 (48‒72)	10.9	‒	KPS > 60	100%	≥3; 100%
Dengina, 2019	Phase Ib	17	54.5 ± 27.5	8	‒	100% KPS ≥80	64.7%	≤1; 35%
Gebbia, 2020	Retrospective	28	64 (40‒74)	‒	‒	100% ECOG 0−1	82%	≤3; 100%
Hammers, 2020 (RADVAX)	Phase II	25	‒	‒	Favorable 8% Intermediate 80% Poor 12%	‒	68%	≥2; 100%
Franzese, 2021	Retrospective	78	66.6 (30‒86)	‒	‒	98% ECOG 0−1	‒	≤5; 80%
Cheung, 2021	Phase II	37	62 (58‒67)	‒	Favorable 32% Intermediate 68%	‒	‒	≤3; 100%
Kroeze, 2021	Retrospective	53	61 (38‒84)	12	‒	89% ECOG 0−1	‒	≤5; 58%
Liu, 2021	Retrospective	42	53 (24‒75)	13.7	Favorable 26.2% Intermediate 52.4% Poor 21.4%	57.1% ECOG 0−1	85.7%	≤5; 28.6%
Hannan, 2022	Phase II	20	60.5 (56‒65)	10.4	Favorable 25% Intermediate 75%	‒	60%	≤5; 40%
Li, 2022	RCT	22	60.94 ± 13.67	‒		100% ECOG ≤2	‒	≤5; 100%
Ma, 2022	Retrospective	35	63 (32‒82)	17	Favorable 17.1% Intermediate 51.4% Poor 37.1%	100% ECOG 0−1	62.9%	≤3; 80%
Masini, 2022 (NIVES)	Phase II	69	66 (43‒84)	26	Favorable 26% Intermediate 65% Poor 9%	100% ECOG 0−1	77%	≥3; 51%
Siva, 2022 (RAPPORT)	Phase I/II trial	30	62 (47‒80)	28	Intermediate 44% Favorable 56%	100% ECOG 0−1	67%	≤5; 100%
Onal, 2023	Retrospective	42	65 (38‒81)	62.3	‒	‒	50.0%	≤5; 100%
Liu, 2023 (abstract)	Phase II	15	‒	‒	‒	‒	‒	‒

Abbreviations: ECOG, Eastern Cooperative Oncology Group; IMDC, International Metastatic Renal Cell Carcinoma Database Consortium; KPS, Karnofsky performance status;RCT, Randomized controlled trial.

The treatment characteristics are described in Table 2. Eight studies included only the extracranial lesions, while eight studies included both intracranial and extracranial lesions. SBRT was prescribed at doses ranging from 5‐20 Gy × 1‒10 fractions for body lesions and 15−50 Gy × 1‒5 fractions for brain lesions This treatment was commonly combined with first‐ or second‐line systemic therapy for either ablation or palliative purposes. Among them, SBRT was concurrent with TKI (five studies) or ICI (five studies) alone or in combination (one study) for all participants. Treatment regimens in five studies were mixed, with participants treated with either SBRT + TKI, SBRT + ICI, or SBRT + mammalian target of rapamycin signaling pathway inhibitor. Sunitinib and pazopanib were commonly used TKIs. Nivolumab, ipilimumab, and pembrolizumab were commonly used ICIs.

**TABLE 2:** Treatment characteristics and main outcomes of included studies.

Author, year	Treating lesions, site (no.)	SBRT schedule	Concurrent systemic therapy lines	Systemic therapy	LCR	ORR	PFS	OS
Oligometastatic setting								
Miller, 2016	Extracranial (‒)	10‒18 Gy/1 fx 21–24 Gy/3 fx	Main first line	TKI (sunitinib, axitinib, pazopanib, sorafenib)	1‐yr 96%	‒	‒	Median OS: 18 mo
Li, 2022	Extracranial and intracranial (‒)	50 Gy/5 fx	Second line 100%	ICI (nivolumab + ipilimumab)	‒	59.09%	Median PFS: 28.1 mo	‒
Ma, 2022	Extracranial (50 lesions)	40‒50 Gy/5 fx 6−8 Gy × 3‒5 fx 50–60 Gy/20‒25 fx	First line 40% Second line 60%	TKI 88.6% ICI 28.6%	‒	‒	1‐yr 41.8% 3‐yr 27.9% Median PFS: 11.3 mo	1‐yr 78.3% 3‐yr 48.2% Median OS: 29.7mo
Siva, 2022 (RAPPORT)	Extracranial (83 lesions)	20 Gy/1 fx	First line 93%	ICI (pembrolizumab)	‒	63%	1‐yr 60% 2‐yr 45%	1‐yr 90% 2‐yr 74%
Onal, 2023	Extracranial and intracranial (96 lesions)	16 or 18 Gy/1 fx 20 or 27 Gy/2 or 3 fx 60 Gy/3 or 4 fx 27−30 Gy/3 or 4 fx 35 Gy/5 fx Brain: 18−20 Gy/1 fx; 24 or 25 Gy/3‒5 fx	First line 100%	TKI (sunitinib, pazopanib, axitinib)	2‐yr 94.1%	‒	2‐y 51.3% Median PFS: 25.7 mo	2‐yr 58.0% Median OS: 30.5 mo
Liu, 2023 (abstract)	Extracranial (36 lesions)	5‒8 Gy × 3−10 fractions	‒	ICI (tislelizumab)	‒	33.30%	‒	‒
Oligoprogressive setting								
Dengina, 2019	Extracranial (17 lesions)	50 Gy/10 fx	Second line 100%	TKI 35% (sunitinib) ICI 29% (nivolumab) mTORi 6% (everolimus, temsirolimus)	‒	‒	‒	‒
Gebbia, 2020	Extracranial and intracranial (28 lesions)	Body: 5‒10 Gy × 5‒10 fx Brain: 21 Gy/1 fx; 27 Gy/3 fx	Second line 100%	TKI (pazopanib)	‒	‒	Median PFS: 4.55 mo	‒
Franzese, 2021	Extracranial and intracranial (‒)	≥5 Gy × 1−10 fx	First line 44.1% Second line 35.9%	TKI 77% (sunitinib, pazopanib) ICI 18% (nivolumab)	1‐yr 80.9% 2‐yr 77.2% 3‐yr 57.5%	‒	1‐yr 75.7% 2‐yr 61.1% 3‐yr 47.2%	1‐yr 90.7% 2‐yr 82.6% 3‐yr 77.7%
Cheung, 2021	Extracranial and intracranial (57 lesions)	48‒60 Gy/3‒8 fx 30–60 Gy/3‒6 fx 30–40 Gy/5 fx 18–40 Gy/1‒5 fx Brain: 15–30 Gy/1‒5 fx	First line 89.2% Second line 10.8%	TKI (sunitinib, pazopanib)	1‐yr 93%	‒	Median PFS: 9.3 mo	1‐yr 92%
Hannan, 2022	Extracranial and intracranial (37 lesions)	≥25 Gy/1 fx ≥36 Gy/3 fx ≥40 Gy/5 fx	First line 20% Second line 50%	TKI 40% ICI 40% ICI + TKI 15% mTORi + TKI 5%	100%	‒	Median PFS: 11.1 mo	
Polymetastatic setting								
De Wolf, 2017	Extracranial (13 lesions)	24 Gy/3 fx 30 Gy/3 fx 36 Gy/3 fx	‒	TKI (pazopanib)	1‐yr 83%	‒	1‐yr 28% Median PFS: 6.7 mo	38%
Hammers, 2020 (RADVAX)	Extracranial (‒)	50 Gy/5 fx	First line 60% Second line 20% ≥Second line 20%	ICI (nivolumab + pembrolizumab)	‒	56%	1‐y 36% Median PFS: 8.2mo	‒
Kroeze, 2021	Extracranial and intro (128 lesions)	Brain: 18−30 Gy/1 fx Body: 24 Gy/3 fx	Median second lines	TKI 60% ICI 32% mTORi 8%	1‐yr 75%	‒	1‐yr 25% ICI: median 11.6 mo TKI: median 5.4 mo	1‐yr 71% ICI: median 19.5 mo TKI: median 18.3 mo
Liu, 2021	Extracranial and intracranial (71 lesions)	30–45 Gy/5 fx	Second line 85.7% Third line 14.3%	TKI + ICI	1‐yr 98.2%	71.80%	Median PFS: 13.2 mo	Median OS: 38.5 mo
Masini, 2022 (NIVES)	Extracranial (‒)	30 Gy/3 fx	Second line 81% Third line19%	ICI (nivolumab)	‒	17%	Median PFS: 5.6 mo	Median OS: 20 mo

Abbreviations: fx, fractions; ICI, immune checkpoint inhibitor; LCR, local control rate; mo, months; mTORi, mammalian target of rapamycin signaling pathway inhibitor; ORR, objective response rate; OS, overall survival; PFS, progression‐free survival; SBRT, stereotactic body radiation therapy; TKI, tyrosine kinase inhibitors; yr, year.

### 2.2 Local control rate

**FIGURE 1:**
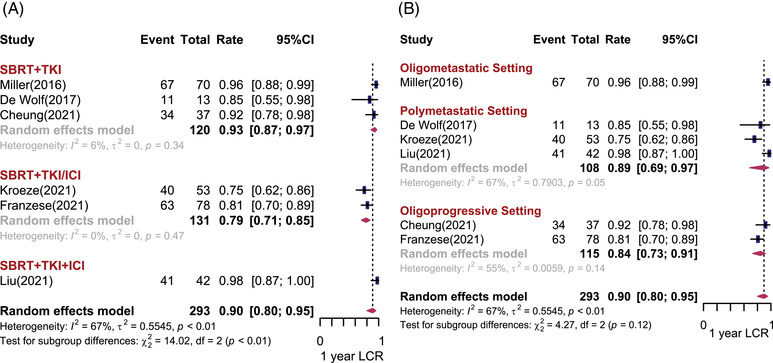
One‐year local control rates (LCRs) after stereotactic body radiotherapy (SBRT) combined with systemic therapy for metastatic renal cell carcinoma. Forest plots depicting weighted random‐effect estimates, 95% confidence intervals (CIs), and heterogeneity for local control after SBRT combined with systemic therapy for metastatic renal cell carcinoma. Subgroup analyses were conducted according to different combination regimens (A) and disease settings (B).

Six studies including 293 patients reported the 1‐year LCR (1‐yr LCR).^8^, ^9^, ^12^, ^13^, ^14^, ^15^ Under the random effects model, the pooled effect size for 1‐yr LCR was 90% (95% confidence interval [CI]: 80%−95%, *I* ^2^ = 67%). The non‐negligible heterogeneity among studies may be attributed to different treatment regimens. In subgroup analysis, the estimated 1‐yr LCR was 93% (95% CI: 87%−97%, *I* ^2^ = 6%) and 79% (95% CI: 71%−85%, *I* ^2^ = 0%) in the SBRT + TKI group and the SBRT + TKI/ICI group, respectively. The SBRT + TKI + ICI group yielded the highest LCR (98%, 95% CI: 87%−100%) compared with other subgroups (*p* < 0.01), although only one study was included (Figure 1A). Regarding the different disease settings (Figure 1B), there was no statistically significant difference in the 1‐yr LCR among patients with oligometastatic (96%, 95% CI: 88%−99%) and oligoprogressive (89%, 95% CI: 69%−97%) or polymetastatic mRCC (84%, 95% CI: 73%−91%). There was no difference between whether the brain lesions were included, and the study design did not affect 1‐yr LCR (Figure S2). However, there was statistically significant publication bias for LCR (Egger test: *p* = 0.05).

For long‐term outcomes, Onal et al.^21^ applied SBRT concurrently with first‐line TKI systemic therapy for oligometastatic mRCC with intracranial lesions, and the 2‐yr LCR reached 94.1%. In a retrospective study conducted by Franzese et al.^13^ including patients with oligoprogressive mRCC, SBRT was administered in first‐ or second‐line TKI/ICI treatment of progressive lesions without systemic therapy withdrawal. The reported 2‐yr and 3‐yr LCRs were 77.2% and 57.5%, respectively.

### 2.3 Objective response rate

**FIGURE 2:**
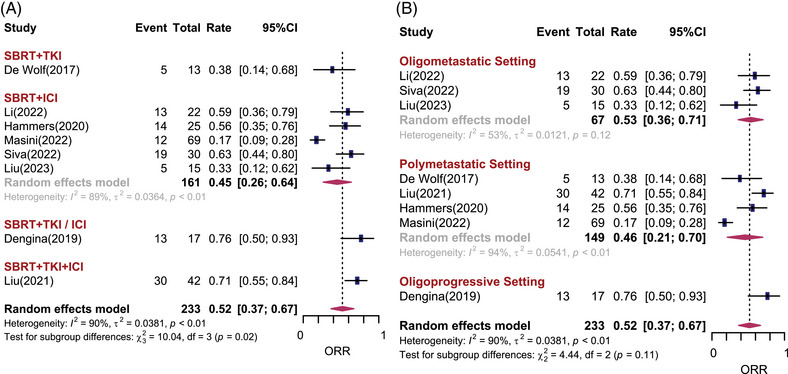
Objective response rates (ORRs) after stereotactic body radiotherapy (SBRT) combined with systemic therapy for metastatic renal cell carcinoma. Forest plots depicting weighted random‐effect estimates, 95% confidence intervals (CIs), and heterogeneity for objective response rates after SBRT combined with systemic therapy for metastatic renal cell carcinoma. Subgroup analyses were conducted according to different combination regimens (A) and disease settings (B).

Eight studies including 233 patients reported objective response rate (ORR). In total, the estimated ORR under the random effects model was 52% (95% CI: 37%−67%, *I* ^2^ = 90%). There was statistically significant difference among different treatment subgroups (*p* = 0.02). For patients prescribed SBRT combined with TKI, ICI, TKI/ICI and TKI + ICI, the estimated ORRs were 38%, 45%, 76%, and 71%, respectively (Figure 2A). There was no difference in ORR among the different disease settings. It seems that studies in which both brain and body lesions were treated had better ORR than those in which only extracranial lesions were treated (68% vs. 47%, *p* = 0.06). One retrospective study significantly reported a higher ORR than prospective studies (Figure S2). There was no statistically significant publication bias for ORR (Egger test: *p* = 0.13).

### 2.4 Progression‐free survival and overall survival

**FIGURE 3:**
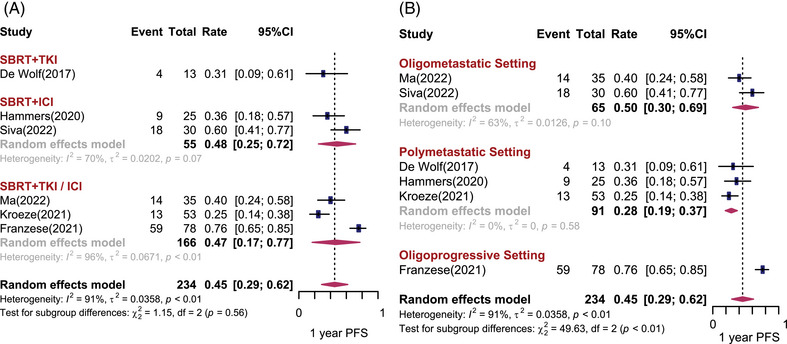
One‐year progression‐free survival (PFS) rates after stereotactic body radiotherapy (SBRT) combined with systemic therapy for metastatic renal cell carcinoma. Forest plots depicting weighted random‐effect estimates, 95% confidence intervals (CIs), and heterogeneity for local control after SBRT combined with systemic therapy for metastatic renal cell carcinoma. Subgroup analyses were conducted according to different combination regimens (A) and disease settings (B).

**FIGURE 4:**
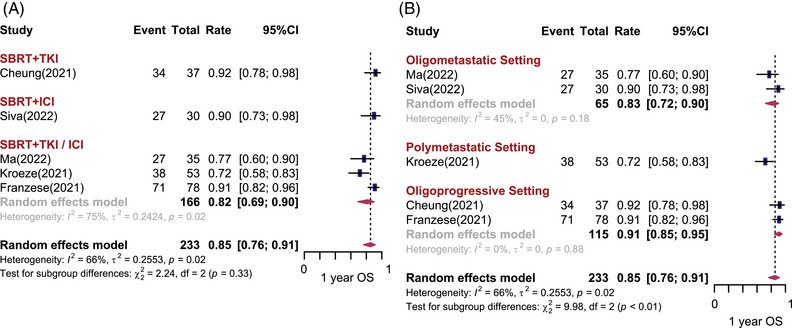
One‐year overall survival (OS) rates after stereotactic body radiotherapy (SBRT) combined with systemic therapy for metastatic renal cell carcinoma. Forest plots depicting weighted random‐effect estimates, 95% confidence intervals (CIs), and heterogeneity for local control after SBRT combined with systemic therapy for metastatic renal cell carcinoma. Subgroup analyses were conducted according to different combination regimens (A) and disease settings (B).

Six studies (*n* = 234) and five studies (*n* = 233) reported 1‐year PFS (1‐yr PFS) rates and 1‐year OS (1‐yr OS) rates, respectively. The pooled results are shown in Figures 3 and 4. Overall, the pooled 1‐yr PFS and 1‐yr OS rates was 45% (95% CI: 29%−62%, *I* ^2^ = 91%) and 85% (95% CI: 76%−91%, *I* ^2^ = 66%), respectively. No significant difference in 1‐yr PFS and 1‐yr OS was observed among different treatment combinations. The 1‐yr PFS was 31% (95% CI: 9%−61%) for SBRT combined with TKI, and 48% (95% CI: 25%−72%, *I* ^2^ = 70%) for SBRT combined with ICI. The 1‐yr OS was 92% (95% CI: 78%−98%) for SBRT combined with TKI, and 90% (95% CI: 73%−98%) for SBRT combined with ICI. Heterogeneity can be partially explained by disease settings. There was statistically significant difference in 1‐yr PFS and 1‐yr OS among the different disease settings (*p* < 0.01). For patients with limited metastases, the estimated 1‐yr PFS was 50% (95% CI: 30−69%, *I* ^2^ = 63%) and 1‐yr OS was 83% (95% CI: 72%−90%, *I* ^2^ = 45%). However, when the number of metastatic sites in a patient exceeded five, the estimated 1‐yr PFS and 1‐yr OS dropped to 28% (95% CI: 19%−37%, *I* ^2^ = 0%) and 72% (95% CI: 58%−83%), respectively. Unexpectedly, studies that included both brain and body metastatic lesions were associated with a preferable 1‐yr PFS and 1‐yr OS (*p* < 0.05) (Figure S2). There was no statistically significant publication bias for PFS (Egger test: *p* = 0.36) and OS (Egger test: *p* = 0.14).

Several studies have reported the long‐term PFS and OS rates. One phase I/II clinical trial included 30 participants with oligometastatic mRCC. Most of the participants were at IMDC favorable risk. SBRT (20 Gy/1 fx) combined with first‐line ICI (pembrolizumab) was used. The 2‐yr PFS and OS rates were 45% and 74%, respectively. In a retrospective study, SBRT was prescribed concurrent with first‐line TKI systemic therapy for oligometastatic mRCC. The 2‐yr PFS rate was 51.3% and 2‐yr OS rate was 58.0%, with a median PFS and OS of 25.7 months (95% CI: 15.1−34.4) and 30.5 months (95% CI: 15.9−43.1), respectively.^21^ Another retrospective study evaluating patients with mRCC who received SBRT with unchanged first‐ or second‐line systemic therapy for progressive lesions reported 2‐yr PFS and OS rates of 61.1% and 82.6%, respectively, and 3‐yr PFS and 3‐yr OS rates of 47.2% and 77.7%, respectively.^13^ Ma et al.^18^ included 35 mRCC patients and the majority of them had IMDC intermediate/poor risk disease (88.5%). Eighty percent of them have less than three metastases; 88.6% of them received SBRT combined TKI therapy, and 28.6% of them received combined ICI therapy. The 3‐yr PFS and OS rates were 27.9% and 48.2%, respectively. The median PFS ranged from 5.6 to 28.1 months and the median OS ranged from 18.0 to 38.5 months.

### 2.5 Toxicity

**FIGURE 5:**
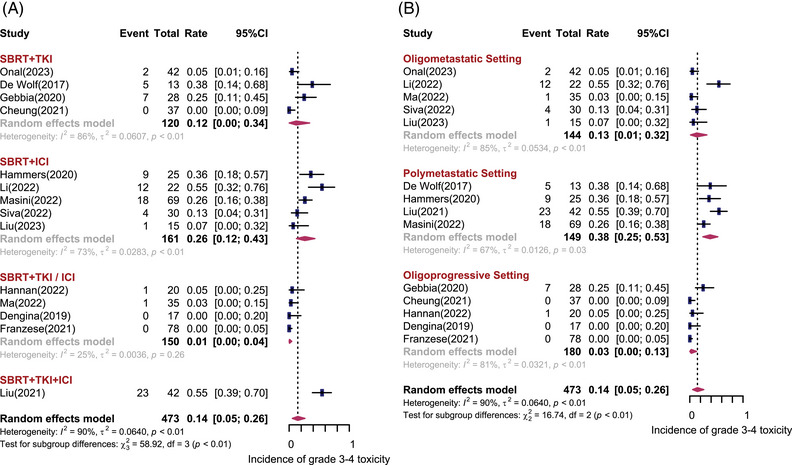
Grade 3−4 toxicity incidence after stereotactic body radiotherapy (SBRT) combined with systemic therapy for metastatic renal cell carcinoma. Forest plots depicting weighted random‐effect estimates, 95% confidence intervals (CIs), and heterogeneity for grade 3−4 toxicity incidence after SBRT combined with systemic therapy for metastatic renal cell carcinoma. Subgroup analyses were conducted according to different combination regimens (A) and disease settings (B).

Fourteen studies including 473 patients depicted the treatment‐related adverse events (AEs). Figure 5 shows the forest plot of grade 3−4 treatment‐related AEs. Overall, the pooled incidence of grade 3−4 AEs was 14% (95% CI: 5%−26%, *I* ^2^ = 90%). The most common types of 3−4 AEs were hypertension, fatigue, gastrointestinal‐related symptoms (diarrhea), liver toxicity (elevated alanine aminotransferase and/or aspartate transaminase), and blood toxicity (anemia). As shown in Figure 5A, patients treated with SBRT concurrent with ICI had higher rates of grade 3−4 AEs compared with SBRT with TKI (26% vs. 12%). Regarding the disease settings (Figure 5B), grade 3−4 AEs were most commonly observed in patients with polymetastatic disease, with the incidence of 38% (95% CI: 25%−53%, *I* ^2^ = 67%), while only 13% (95% CI: 1%−32%, *I* ^2^ = 85%) of grade 3−4 AEs were found in oligometastatic setting. The reported incidence of grade 1−2 AEs ranged from 12% to 100%. Patients treated with SBRT concurrent with ICI experienced higher rates of grade 1−2 AEs. The types of grade 1−2 AEs are summarized in Table 3. No grade 5 AEs were observed.

**TABLE 3:** Treatment‐related adverse events (AEs) of included studies.

Author, year	Incidence (1‒2 grade)	AEs	Incidence (3‒4 grade)	AEs
Miller, 2016	‒	‒	‒	‒
De Wolf, 2017	‒	‒	38%	Hypoglycemia; increased ALT/AST; hypertension
Dengina, 2019	12%	Oesophagitis; skin erythema	0%	‒
Gebbia, 2020	‒	Thyroid changes; diarrhea; fatigue; anemia; liver toxicity; mucositis pneumonitis; esophagitis	25%	Hypertension; liver toxicity; diarrhea; pneumonitis
Hammers, 2020 (RADVAX)	100%	Fatigue; skin, respiratory, gastrointestinal, renal, endocrine related; arthritis	36%	Gastrointestinal related
Franzese, 2021	‒	Pain; nausea; cough; dysphagia; dyspnea; diarrhea	0%	‒
Cheung, 2021	19%	Nausea/vomiting; dyspnea or cough; pneumonitis; bone/chest wall pain	0%	‒
Kroeze, 2021	‒	‒	‒	Dysarthria; incomplete paresis; headache; vertigo
Liu, 2021	‒	‒	54.80%	Hypertension; fatigue; proteinuria; anemia
Hannan, 2022	93.3%	Abdominal pain; increased ALT/AST; epigastric pain; fatigue; lymphocyte count decreased; nausea; vomiting	5%	Colitis
Li, 2022	‒	‒	54.50%	‒
Ma, 2022	‒	Gastrointestinal related; anemia	2.90%	Anemia
Masini, 2022 (NIVES)	‒	Rash; fatigue; pneumonitis; endocrine disorders	26%	Diarrhea; hyperlipasemia; hyperamylasemia; fatigue
Siva, 2022 (RAPPORT)	63%	‒	13%	Pneumonitis; dyspnea; increased ALT/AST
Onal, 2023	‒	Esophagitis; pain; diarrhea; dermatitis; anemia; fatigue; liver toxicity	4.80%	Diarrhea; hypertension
Liu, 2023 (Abstract)	‒	Fatigue; hypothyroidism; pneumonia	6.70%	Hematologic toxicity

Abbreviations: ALT, alanine aminotransferase; AST, aspartate transaminase.

## 3 DISCUSSION

Overall, metastasis‐directed SBRT concomitant with systemic therapies, including TKIs and ICIs, is safe and effective in the management of mRCC. SBRT concomitant with TKI + ICI yielded the best 1‐yr LCR and 1‐yr ORR for mRCC patients. Nevertheless, this therapeutic strategy had the greatest toxicity, not surprisingly. SBRT concomitant with ICI seems to be more effective in producing better treatment response than combined with TKI. Regarding the survival benefits, there was a trend toward better 1‐yr PFS for patients receiving ICI therapy compared to TKI, whereas this difference was not statistically significant. And there was no statistically significant difference between treatment regimens on the OS. In terms of toxicity, SBRT concomitant with ICI significantly elevated 3−4 AEs compared with TKI. Of note, from the other perspective, disease settings did not significantly affect 1‐yr LCR and 1‐yr ORR, but patients with polymetastatic disease had significantly worst survival outcomes regardless of treatment combinations. A previous study systematically reviewed and addressed the efficacy and safety of SBRT in combination with drugs in mRCC^22^; however, to the best of our knowledge, our study is the first to conduct meta‐analyses and address combination strategies within different metastasis contexts.

Research on the treatment approaches involving the combination of TKIs with ICIs has been actively pursued and has recently yielded promising outcomes in advanced kidney cancer. A phase III CLEAR trial (NCT02811861) enrolled treatment‐naïve patients with advanced RCC with a clear cell component. Investigators presented that the median OS was 47.9 (40.5‐NE) months and the median PFS was 22.1 (16.6‒27.6) months for intermediate/poor risk patients receiving TKI lenvatinib in combination with PD‐1 inhibitor pembrolizumab. Grade 3 or higher treatment‐related AEs occurred in 74.1% of patients.^23^ In an additional phase III clinical trial, KEYNOTE‐426 (NCT02853331), the trial investigated the safety and efficacy of combining pembrolizumab with another TKI, axitinib (Inlyta), in patients with treatment‐naïve locally advanced or metastatic ccRCC. Findings from the 5‐year analysis of KEYNOTE‐426 demonstrated that at a median follow‐up of 67.2 months (range 60.0‒75.0), the median PFS was 15.7 months (95% CI: 13.6‒20.2). The ORR was 60.6% with an 11.6% complete response rate. The median OS was 47.2 months (95% CI: 43.6‒54.8).^24^ Multiple studies that presented median survival outcomes are summarized in Table 2, and these results have led to the concerns and discussion about whether SBRT plays a role in prolonging PFS/OS in mRCC patients treating with systemic therapy. More prospective research and clinical trials are needed to compare the efficacy and safety of SBRT + TKI or/and ICI versus TKI or/and ICI.

In theory, the immunogenic effects of radiation could be impacted by the dosage and fractionation of radiation administered. A single dose of radiotherapy versus multiple fractionated doses may lead to notably distinct gene expression profiles in cancer cells.^25^ Furthermore, given the sensitivity of lymphocytes to irradiation, the repetitive administration of cytotoxic radiation doses may result in the depletion of migrating immune effector cells such as CD8+ T cells, CD4+ T cells, and NK cells, all of which are vital for immunotherapy. In contrast, a single dose of radiotherapy does not have the same effect.^26^ SBRT represents a radiation approach usually administered in a single session or a limited number of fractions, delivering ablative doses to small target volumes. Therefore, this technique may evoke a stronger immune response and holds greater promise for combination with immunotherapy compared to conventional radiotherapy.^26^

In clinical practice, prior studies have furnished evidence supporting the utilization of SBRT in the oligometastatic context.^27^, ^28^ The pooled evidence of this study also demonstrated that using SBRT combined with systemic therapy in the oligometastatic setting is well tolerated and effective. The use of SBRT has also been considered in the oligoprogressive setting, with the aim of delaying the time of systemic therapy switch and sparing a line of treatment.^29^ Prior researches have shown that employing SBRT can notably prolong the interval before transitioning to subsequent lines of drug therapy, extend the duration of systemic therapy before progression, and reduce the incidence of metastases during oligoprogression. These factors are associated with longer PFS and benefit most from utilizing SBRT in the context of oligoprogression.^12^, ^13^, ^16^ In our study, SBRT could also induce promising treatment response and local tumor control in progressive lesions.

Previous research examined the effectiveness of SBRT in patients with mRCC who were concurrently undergoing targeted therapy or immunotherapy.^14^ In this study, 58% of the participants belong to oligometastatic or oligoprogressive setting, and 42% of them have more than five metastases. Patients with oligometastatic disease had a significantly better PFS compare to those with more than five lesions (Hazard Ratio: 0.34, *p* = 0.003), with a median of 11.6 month versus 3.3 months, which is consistent with our results. Intriguingly, it has recently been suggested that SBRT may have the capability to augment the impact of immunotherapy in individuals with polymetastatic mRCC. A recent phase II clinical trial (RADVAX) has provided encouraging results of patients with polymetastatic mRCC who received SBRT (50 Gy/5 fx) concurrent with dual immune checkpoint blockade (nivolumab + ipilimumab). The majority of patients were intermediate risk for IMDC. The most commonly radiated site was the lung. The overall response rate was 56%, and the median PFS was 8.2 months. However, it was unclear whether these findings represent a true abscopal effect. On the contrary, a phase II trial (NIVES) also included 69 patients with polymetastatic mRCC. The majority of patients were diagnosed with intermediate risk for IMDC. SBRT was administered at 30 Gy in three fractions with nivolumab. Unfortunately, this study did not provide positive evidence to support that nivolumab in combination with SBRT provides an additional benefit in pretreated mRCC patients, with an ORR of 17% and a median PFS of 4.1 months. The disparities observed between these two trials have prompted inquiries regarding the timing of SBRT and the selection of patients. In the RADVAX trial, SBRT was mostly administered concurrently with first‐line systemic therapy, while in the NIVES trial, the majority of SBRT was combined with second‐line systemic therapy. Previous studies demonstrated that the number of prior lines of systemic therapy was a prognostic factor for patients treated with stereotactic radiosurgery.^30^ Therefore, early use of SBRT is encouraged. In our study, the pooled ORR in the polymetastatic mRCC setting was 46%, which was not inferior to oligo/oligoprogressive setting. However, the 1‐yr PFS was significantly poorer. Furthermore, more data are needed to support the use of SBRT in the polymetastatic mRCC setting.

Our work has limitations. First, due to the lack of access to individual patient information, it was not possible to adjust for patient‐specific covariates. Second, the studies included reported outcomes in various formats, resulting in only a portion of these outcomes being suitable for meta‐analyses. Additionally, the relatively small number of studies included in subgroup analyses warrants caution when interpreting these results. Furthermore, some studies, particularly those with a retrospective design, did not distinguish outcomes based on concurrent systemic therapies, leading to mixed results. Third, our analyses incorporated both retrospective and prospective studies, with variations observed in treatment sites, systemic drugs, SBRT regimens, and concurrent timing, potentially contributing to heterogeneity among the studies.

In conclusion, SBRT applied during systemic therapy is safe and effective in the management of mRCC, especially for patients with a low metastatic tumor burden, although how best to implement this strategy is still unclear. Future basic researches are needed to help us understand the biology, provide stronger rationale for clinical practice, and guide the design of future clinical trials.

## 4 MATERIALS AND METHODS

This study adhered to the Preferred Reporting Items for Systematic Review and Meta‐analysis (PRISMA) statement,^31^ and followed the systematic review methodology outlined by the European Association of Urology.^32^ The protocol of this study was PROSPERO registered (registration number: CRD42023425718).

### 4.1 Search strategy and eligibility criteria

We systematically searched PubMed and American Society of Clinical Oncology annual meeting abstracts up to February 2023 to identify potentially relevant studies. The following search strings were used: (stereotactic [Title/Abstract]) AND (RCC [MeSH Terms]). The inclusion criteria were defined using the Population, Intervention, Control, Outcome, Study Design (PICOS) approach: (P) patients with mRCC; (I) received metastasis‐directed SBRT, defined as high dose per fraction (typically ≥5 Gy per fraction, highly conformal radiotherapy delivered as a single fraction to an intracranial site, or small number of fractions to an extracranial site); (C) either no control group or a multi‐arm study where SBRT was administered; (O) at least reporting one or more tumor control outcomes or any AEs; and (S) a prospective or retrospective design.

### 4.2 Study selection and data extraction

Two independent reviewers screened all titles and abstracts and evaluated the full text for eligibility. Data extraction was carried out independently by two reviewers, with any discrepancies resolved by a third reviewer. The selection process was presented by PRISMA flowchart (Figure S1). The following data were extracted from included studies: author, year of publication, study design, sample size, age of participants, median follow‐up duration, IMDC risk, performance status, primary tumor surgery, and number of metastases. Oligometastases represent a clinical situation in patients with limited metastases (≤5). Otherwise, defines as polymetastatic disease. Oligoprogression represents that five or fewer metastases have progressed, while other sites are controlled under systemic therapy.^33^ The SBRT schedule and concurrent systematic treatment lines and strategies were also extracted. The relative oncological outcomes and toxicity were recorded.

### 4.3 Outcomes

The primary outcomes included 1‐yr LCR, ORR, 1‐yr PFS, and 1‐yr OS. Few studies have reported the long‐term outcomes. Meta‐analysis was performed when these data were available; otherwise, the results were systematically reviewed. The secondary outcome was incidence of any grade 3−4 acute or late AEs according to the Common Terminology Criteria for Adverse Events (version 3.0 or 4.0).

### 4.4 Risk of bias assessment

The risk of bias (RoB) was independently assessed by two reviewers, and any discrepancies were solved by a third reviewer. According to the EAU methodology, five aspects were considered (Table S1). If the answer to all five questions was “yes,” the study was considered to have a “low” RoB. If the answer to any question was “no,” the study was considered to have a “high” RoB. All 15 full‐length articles were undertaken RoB assessment except one meeting abstract.

### 4.5 Synthesis of results and statistical analysis

The primary outcomes are reported as rates with 95% CIs. Random effects model or fixed effects model was applied according to the between‐study heterogeneity.^34^ Heterogeneity across studies was formally tested according to chi‐square (*p* < 0.05) and the *I* ^2^ statistic. Egger test of funnel plot symmetry was conducted to evaluate publication bias.^35^ A *p*‐value < 0.1 represents a statistically significant risk of publication bias. Additionally, to evaluate whether concurrent systematic treatment strategies, disease settings, treated metastatic sites, and study design could explain the heterogeneity of treatment efficacy and AEs between trials, pre‐planned subgroup analyses were conducted for all of our outcomes. In terms of survival outcomes (except our pooled outcomes), a narrative synthesis was performed due to the different reporting forms among included studies. A *p*‐value < 0.05 was considered as statistically significant. All statistical analyses were performed using the “meta” and “metafor” packages in R 4.1.2 (R project).

## AUTHOR CONTRIBUTIONS

*Study concept and design*: P.T. and S.Z. *Manuscript writing*: S.Z. *Study selection and data extraction*: Y.L. and N.X. *Data analyses*: X.X. and S.Z. *Review/editing of manuscript before submission*: P.T. and Q.W. All authors have read and approved the final manuscript.

## CONFLICT OF INTEREST STATEMENT

The authors declare they have no conflicts of interest.

## ETHICS STATEMENT

Not applicable.

## Supporting information

Supporting Information

## Data Availability

All the data can be found in the manuscript or Supporting Information.

## References

[1] Sung H , Ferlay J , Siegel RL , et al. Global cancer statistics 2020: GLOBOCAN estimates of incidence and mortality worldwide for 36 cancers in 185 countries. CA Cancer J Clin. 2021;71(3):209‐249. doi:10.3322/caac.21660 33538338

[2] Bukavina L , Bensalah K , Bray F , et al. Epidemiology of renal cell carcinoma: 2022 update. Eur Urol. 2022;82(5):529‐542. doi:10.1016/j.eururo.2022.08.019 36100483

[3] Gupta K , Miller JD , Li JZ , Russell MW , Charbonneau C . Epidemiologic and socioeconomic burden of metastatic renal cell carcinoma (mRCC): a literature review. Cancer Treat Rev. 2008;34(3):193‐205. doi:10.1016/j.ctrv.2007.12.001 18313224

[4] Siva S , Kothari G , Muacevic A , et al. Radiotherapy for renal cell carcinoma: renaissance of an overlooked approach. Nat Rev Urol. 2017;14(9):549‐563. doi:10.1038/nrurol.2017.87 28631740

[5] Stenman M , Sinclair G , Paavola P , Wersäll P , Harmenberg U , Lindskog M . Overall survival after stereotactic radiotherapy or surgical metastasectomy in oligometastatic renal cell carcinoma patients treated at two Swedish centres 2005–2014. Radiother Oncol. 2018;127(3):501‐506. doi:10.1016/j.radonc.2018.04.028 29754859

[6] Zaorsky NG , Lehrer EJ , Kothari G , Louie AV , Siva S . Stereotactic ablative radiation therapy for oligometastatic renal cell carcinoma (SABR ORCA): a meta‐analysis of 28 studies. Eur Urol Oncol. 2019;2(5):515‐523. doi:10.1016/j.euo.2019.05.007 31302061

[7] Hunter GK , Balagamwala EH , Koyfman SA , et al. The efficacy of external beam radiotherapy and stereotactic body radiotherapy for painful spinal metastases from renal cell carcinoma. Pract Radiat Oncol. 2012;2(4):e95‐e100. doi:10.1016/j.prro.2012.01.005 24674192

[8] Miller JA , Balagamwala EH , Angelov L , et al. Spine stereotactic radiosurgery with concurrent tyrosine kinase inhibitors for metastatic renal cell carcinoma. J Neurosurg Spine. 2016;25(6):766‐774. doi:10.3171/2016.4.SPINE16229 27391397

[9] De Wolf K , Rottey S , Vermaelen K , et al. Combined high dose radiation and pazopanib in metastatic renal cell carcinoma: a phase I dose escalation trial. Radiat Oncol. 2017;12(1):157. doi:10.1186/s13014-017-0893-x 28938918 PMC5610443

[10] Dengina N , Mitin T , Gamayunov S , Safina S , Kreinina Y , Tsimafeyeu I . Stereotactic body radiation therapy in combination with systemic therapy for metastatic renal cell carcinoma: a prospective multicentre study. ESMO Open. 2019;4(5):e000535. doi:10.1136/esmoopen-2019-000535 31673426 PMC6802957

[11] Gebbia V , Girlando A , DIG A , et al. Stereotactic radiotherapy for the treatment of patients with oligo‐progressive metastatic renal cell carcinoma receiving vascular endothelial growth factor receptor tyrosine kinase inhibitor: data from the real world. Anticancer Res. 2020;40(12):7037‐7043. doi:10.21873/anticanres.14730 33288600

[12] Cheung P , Patel S , North SA , et al. Stereotactic radiotherapy for oligoprogression in metastatic renal cell cancer patients receiving tyrosine kinase inhibitor therapy: a phase 2 prospective multicenter study. Eur Urol. 2021;80(6):693‐700. doi:10.1016/j.eururo.2021.07.026 34399998

[13] Franzese C , Marvaso G , Francolini G , et al. The role of stereotactic body radiation therapy and its integration with systemic therapies in metastatic kidney cancer: a multicenter study on behalf of the AIRO (Italian Association of Radiotherapy and Clinical Oncology) genitourinary study group. Clin Exp Metastasis. 2021;38(6):527‐537. doi:10.1007/s10585-021-10131-w 34748125

[14] Kroeze SGC , Fritz C , Schaule J , et al. Stereotactic radiotherapy combined with immunotherapy or targeted therapy for metastatic renal cell carcinoma. BJU Int. 2021;127(6):703‐711. doi:10.1111/bju.15284 33113260

[15] Liu Y , Zhang Z , Liu R , et al. Stereotactic body radiotherapy in combination with non‐frontline PD‐1 inhibitors and targeted agents in metastatic renal cell carcinoma. Radiat Oncol. 2021;16(1):211. doi:10.1186/s13014-021-01937-9 34727963 PMC8561986

[16] Hannan R , Christensen M , Hammers H , et al. Phase II trial of stereotactic ablative radiation for oligoprogressive metastatic kidney cancer. Eur Urol Oncol. 2022;5(2):216‐224. doi:10.1016/j.euo.2021.12.001 34986993 PMC9090939

[17] Li W , Cao Z , Chang P , Zhang B , Li F , Chang D . Clinical efficacy of PD‐1 inhibitors plus split‐course radiotherapy in the first‐line treatment of advanced kidney cancer: a randomized controlled trial. J Oncol. 2022;2022:8100323. doi:10.1155/2022/8100323 35942408 PMC9356868

[18] Ma M‐W , Li H‐Z , Gao X‐S , et al. Outcomes of high‐dose stereotactic ablative radiotherapy to all/multiple sites for oligometastatic renal cell cancer patients. Curr Oncol. 2022;29(10):7832‐7841. doi:10.3390/curroncol29100619 36290896 PMC9600736

[19] Masini C , Iotti C , De Giorgi U , et al. Nivolumab in combination with stereotactic body radiotherapy in pretreated patients with metastatic renal cell carcinoma. Results of the phase II NIVES study. Eur Urol. 2022;81(3):274‐282. doi:10.1016/j.eururo.2021.09.016 34602312

[20] Siva S , Bressel M , Wood ST , et al. Stereotactic radiotherapy and short‐course pembrolizumab for oligometastatic renal cell carcinoma—the RAPPORT trial. Eur Urol. 2022;81(4):364‐372. doi:10.1016/j.eururo.2021.12.006 34953600

[21] Onal C , Oymak E , Guler OC , et al. Stereotactic body radiotherapy and tyrosine kinase inhibitors in patients with oligometastatic renal cell carcinoma: a multi‐institutional study. Strahlenther Onkol. 2023;199(5):456‐464. doi:10.1007/s00066-022-02026-w 36450836

[22] Ingrosso G , Becherini C , Francolini G , et al. Stereotactic body radiotherapy (SBRT) in combination with drugs in metastatic kidney cancer: a systematic review. Crit Rev Oncol Hematol. 2021;159:103242. doi:10.1016/j.critrevonc.2021.103242 33545356

[23] Motzer RJ , Porta C , Eto M , et al. Final prespecified overall survival (OS) analysis of CLEAR: 4‐year follow‐up of lenvatinib plus pembrolizumab (L+P) vs sunitinib (S) in patients (pts) with advanced renal cell carcinoma (aRCC). J Clin Oncol. 2023;41(suppl 16):4502. doi:10.1200/JCO.2023.41.16_suppl.4502

[24] Rini BI , Plimack ER , Stus V , et al. Pembrolizumab plus axitinib versus sunitinib as first‐line therapy for advanced clear cell renal cell carcinoma. J Clin Oncol. 2023;41(suppl 17). doi:10.1200/JCO.2023.41.17_suppl.LBA4501

[25] Tsai MH , Cook JA , Chandramouli GV , et al. Gene expression profiling of breast, prostate, and glioma cells following single versus fractionated doses of radiation. Cancer Res. 2007;67(8):3845‐3852. doi:10.1158/0008-5472.Can-06-4250 17440099

[26] Siva S , MacManus MP , Martin RF , Martin OA . Abscopal effects of radiation therapy: a clinical review for the radiobiologist. Cancer Lett. 2015;356(1):82‐90. doi:10.1016/j.canlet.2013.09.018 24125863

[27] Lehrer EJ , Singh R , Wang M , et al. Safety and survival rates associated with ablative stereotactic radiotherapy for patients with oligometastatic cancer: a systematic review and meta‐analysis. JAMA Oncol. 2021;7(1):92‐106. doi:10.1001/jamaoncol.2020.6146 33237270 PMC7689573

[28] Kroeze SGC , Pavic M , Stellamans K , et al. Metastases‐directed stereotactic body radiotherapy in combination with targeted therapy or immunotherapy: systematic review and consensus recommendations by the EORTC‐ESTRO OligoCare consortium. Lancet Oncol. 2023;24(3):e121‐e132. doi:10.1016/s1470-2045(22)00752-5 36858728

[29] Patel PH , Palma D , McDonald F , Tree AC . The Dandelion dilemma revisited for oligoprogression: treat the whole lawn or weed selectively? Clin Oncol (R Coll Radiol). 2019;31(12):824‐833. doi:10.1016/j.clon.2019.05.015 31182289 PMC6880295

[30] Lanier CM , McTyre E , LeCompte M , et al. The number of prior lines of systemic therapy as a prognostic factor for patients with brain metastases treated with stereotactic radiosurgery: results of a large single institution retrospective analysis. Clin Neurol Neurosurg. 2018;165:24‐28. doi:10.1016/j.clineuro.2017.12.021 29289917

[31] Page MJ , McKenzie JE , Bossuyt PM , et al. The PRISMA 2020 statement: an updated guideline for reporting systematic reviews. BMJ. 2021;372:n71. doi:10.1136/bmj.n71 33782057 PMC8005924

[32] Knoll T , Omar MI , Maclennan S , et al. Key steps in conducting systematic reviews for underpinning clinical practice guidelines: methodology of the European Association of Urology. Eur Urol. 2018;73(2):290‐300. doi:10.1016/j.eururo.2017.08.016 28917594

[33] Weichselbaum RR , Hellman S . Oligometastases revisited. Nat Rev Clin Oncol. 2011;8(6):378‐382. doi:10.1038/nrclinonc.2011.44 21423255

[34] Lau J , Ioannidis JP , Schmid CH . Quantitative synthesis in systematic reviews. Ann Intern Med. 1997;127(9):820‐826. doi:10.7326/0003-4819-127-9-199711010-00008 9382404

[35] Egger M , Davey Smith G , Schneider M , Minder C . Bias in meta‐analysis detected by a simple, graphical test. BMJ. 1997;315(7109):629‐634. doi:10.1136/bmj.315.7109.629 9310563 PMC2127453

