# Supplementary material for: The efficacy and safety of stereotactic body radiotherapy combined with systematic therapy for metastatic renal cell carcinoma: a systematic review and meta‐analysis
Source: MedComm (2020). 2024 Apr 24;5(5):e544. doi: 10.1002/mco2.544 (PMC11042534; doi:10.1002/mco2.544)
Supplement: Supplementary file 1 — Supporting Information [file MCO2-5-e544-s001.docx]

Supplemental materials

**The efficacy and safety of stereotactic body radiotherapy combined with systematic therapy for metastatic renal cell carcinoma: A systematic review and meta-analysis**

Shiyu Zhang^1#^, Xingyu Xiong^1#^, Nan Xie^3#^, Weitao Zheng^1^, Yongjun Li^2^, Tianhai Lin^1﹡^, Qiang Wei^1﹡^, Ping Tan^1﹡^

1. Department of Urology, Institute of Urology, West China Hospital of Sichuan University, Chengdu, 610041, Sichuan, China

2. West China School of Medicine, West China Hospital, Sichuan University, Chengdu, Sichuan, China

3. Emergency Department of West China Hospital, Sichuan University, West China School of Nursing, Institute of Disaster Medicine, Sichuan University; Nursing Key Laboratory of Sichuan Province.

# These authors contribute equally.

#Correspondence to:

Dr. Qiang Wei, Department of Urology, Institute of Urology, West China Hospital of Sichuan University, No. 37, Guoxue Alley, Chengdu, Sichuan, P.R. China; Post Code: 610041; weiqiang163163@163.com, telephone: +86 18980601425.

Dr. Ping Tan, Department of Urology, Institute of Urology, State Key Laboratory of Biotherapy and Cancer Center, West China Hospital of Sichuan University, No. 37, Guoxue Alley, Chengdu, Sichuan, P.R. China; Post Code: 610041; uro_tanping@scu.edu.cn, telephone: +86 18782948613.

Dr. Tianhai Lin, Department of Urology, Institute of Urology, West China Hospital of Sichuan University, No. 37, Guoxue Alley, Chengdu, Sichuan, P.R. China; Post Code: 610041; tlin@wchscu.cn, telephone: +86 18980600969.

**Supplemental Figure 1 The PRISMA flowchart.**

PRISMA = Preferred Reporting Items for Systematic Reviews and Meta-Analyses, SBRT= stereotactic body radiotherapy.

**Supplemental Figure 2** **1-yr local control, objective response rates and 1-yr progression free survival, 1-year overall survival rates after SBRT combined with systemic therapy for metastatic renal cell carcinoma.**

Forest plots depicting weighted random-effect estimates, 95% confidence intervals, and heterogeneity for (A and B) local control, (C and D) objective response rates, (E and F) 1-yr progression free survival, (G and H) 1-year overall survival rates after SBRT combined with systemic therapy for metastatic renal cell carcinoma. Subgroup analyses were conducted according to different radiation sites and study design. CI= confidence interval; LCR= local control rates; ORR= objective response rates; PFS= progression free survival; OS= overall survival;

**Supplemental Figure 3 Grade 3-4 toxicity incidence after SBRT combined with systemic therapy for metastatic renal cell carcinoma.**

Forest plots depicting weighted random-effect estimates, 95% confidence intervals, and heterogeneity for grade 3-4 toxicity incidence after SBRT combined with systemic therapy for metastatic renal cell carcinoma. Subgroup analyses were conducted according to different radiation sites (A) and study design (B). CI= confidence interval;

**Supplemental Figure 1**

**Supplemental Figure 2**.

**Supplemental Figure 3.**

**Supplemental Table 1 Risk of bias assessment of included studies**

| Study(year) | 1 | 2 | 3 | 4 | 5 | RoB |
| --- | --- | --- | --- | --- | --- | --- |
| Miller (2016) | N | Y | Y | Y | Y | High |
| De Wolf (2017) | Y | Y | Y | Y | Y | Low |
| Dengina (2019) | N | Y | Y | N | Y | High |
| Gebbia (2020) | N | Y | Y | Y | Y | High |
| Cheung (2021) | Y | Y | Y | Y | Y | Low |
| Franzese (2021) | N | Y | Y | Y | Y | High |
| Kroeze (2021) | Y | Y | Y | Y | Y | Low |
| Liu (2021) | Y | Y | Y | Y | Y | Low |
| Hannan (2022) | Y | Y | Y | Y | Y | Low |
| Li (2022) | Y | Y | Y | Y | Y | Low |
| Ma (2022) | N | Y | Y | Y | Y | High |
| Masini (2022) | Y | Y | Y | Y | Y | Low |
| Siva (2022) | Y | Y | Y | Y | Y | Low |
| Onal (2023) | N | Y | Y | Y | N | High |

Supplemental Table 1. Risk of Bias of included studies. RoB=Risk of Bias; Y=Yes; N=No.

1.Was there an a priori protocol?

2.Was the total population included or were study participants selected consecutively?

3.Was outcome data complete for all participants and any missing data adequately explained/unlikely to be related to the outcome?

4.Were all prespecified outcomes of interest and expected outcomes reported?

5.Were primary benefit and harm outcomes appropriately measured?

If the answer to all five questions is “yes,” the study is at “low” RoB. If the answer to any question is “no,” the study is at “high” RoB.
